# Comparison of Two Methods for the Determination of the Effects of Ionizing Radiation on Blood Cell Counts in Mice

**Published:** 2012-03

**Authors:** Ana L. Romero-Weaver, Ann R. Kennedy

**Affiliations:** *Department of Radiation Oncology, University of Pennsylvania School of Medicine, Philadelphia, Pennsylvania, USA*

**Keywords:** granulocytes, gamma radiation, proton radiation, white blood cell counts, lymphocytes

## Abstract

A reliable technique is needed to determine the effect of ionizing radiation on white blood cell (WBC) counts. Facilities that utilize automated methods can provide this service. However, utilizing external facilities can introduce additional variables, such as differences between time of sample collection and time of sample processing, which may affect the results. The purpose of the present study was to determine whether an automated method at an external facility can accurately determine radiation-induced changes in total WBC, lymphocyte and granulocyte counts when samples are analyzed at periods of time up to 24 hours after collection and stored either at room temperature or at 4°C. To accomplish this, we compared automated blood cell counts determined at an external facility with our manual blood cell counts processed immediately after sample collection or 24 h after sample collection and stored either at room temperature or 4°C from mice exposed to 2 Gy proton or 2 Gy gamma radiation. Our results show a close correlation and good agreement between the two methods, indicating that neither a delay of 24 hours in sample processing nor storage temperature affected white blood cell counts. Analysis of the effects of radiation on blood cell counts by either manual or automated cell counts revealed a statistically significant decrease in lymphocyte and granulocyte counts at different days post-irradiation, with no statistically significant difference between the methods employed; therefore both manual and automated blood cell counts are reliable methods to determine the effects of ionizing radiation in blood cells.

## INTRODUCTION

The present work was performed as part of a National Space Biomedical Research Institute (NSBRI) supported Center for Acute Radiation Research (CARR) grant. In the CARR grant work, there is particular emphasis on determining the effects of solar particle event (SPE) radiation on white blood cell (WBC) counts, as these cells are known to be particularly sensitive to the cell killing effects of ionizing radiation. To evaluate the effects of radiation on blood cell numbers, reliable and efficient techniques are required. Manual and automated methods are routinely utilized to determine blood cell counts. Manual blood cell counts are based on the staining characteristics exhibited by each blood cell type after being stained with a Romanowsky-type stain such as Wright-Giemsa (1, 2). Several instrument-based systems are currently available for automated blood cell counting (3, 4). These counters are based on impedance and flow cytometry principles (5). Originally, automated counts were limited in their capacity to determine cell morphology and could not accurately differentiate immature or abnormal cells (6, 7). However, current automated counters provide better detection of morphological abnormalities; therefore, their use over manual examination of blood smears has increased considerably in recent years (8, 9). Moreover, there are commercial facilities that provide automated blood cell count services. Using these services in external facilities may improve research laboratory efficiency, thereby freeing research personnel from labor intensive manual cell counting activities so that they can focus on other more important activities and by leveraging the technical expertise and scale of economy in the external facilities specialized in providing such services. However, the use of an automated blood count service in external facilities requires that the blood sample collection and analysis be separated both in time and geographic locations. The difference in test methods (manual counting versus instrument-based automated counting), time and locations (test immediately at the laboratory of study versus testing up to 24 hours later in a remote external facility) could potentially affect the reliability of the results by introducing additional variables. It has been reported that collection and processing methods can affect neutrophil and monocyte counts when sample processing is delayed (10); however, no information is available on the effects that differences between time of sample collection and time of sample processing or storage temperature may cause in samples collected from irradiated animals and processed 24 hours later. To address this, we conducted a study to compare automated differential counts determined at an external facility up to 24 h after sample collection with our in house manual differential counts determined immediately after sample collection or 24 h after sample collection and storage at room temperature or at 4°C from mice exposed to 2 Gy proton radiation or 2 Gy gamma radiation. Our results demonstrated a strong correlation and good agreement between the two methods utilized, indicating that a delay of up to 24 hours between sample collection and sample analysis and storage of samples at either room temperature or 4°C does not affect blood cell counts in samples taken from mice exposed to ionizing radiation. Analysis of the effects of gamma and proton radiation on lymphocytes and granulocytes by manual and automated counts showed a statistically significant decrease in these blood cells at different days post-irradiation with no statistically significant difference between the methods utilized. Therefore, the effects of radiation on blood cells can be accurately determined by either method.

## MATERIAL AND METHODS

### Animals

Female ICR mice aged 4–5 weeks were purchased from Taconic Farms Inc. (Germantown, NY). Animals were acclimated for 7 days at the University of Pennsylvania animal facility. Five animals were housed per cage with *ad libitum* access to water and food pellets. The animal care and treatment procedures were approved by the Institutional Animal Care and Use Committee of the University of Pennsylvania.

### Gamma irradiation

Female mice were restrained in custom designed Plexiglass chambers and exposed to a dose of 2 Gy gamma radiation administered at a dose rate of 0.44 Gy/minute in a (^137^Cs) Gammacell 40 irradiator.

### Proton irradiation

Female mice were restrained in Plexiglass chambers and exposed to a dose of 2 Gy proton radiation administered at a dose rate of 0.5 Gy/minute with a Spread Out Bragg Peak (SOBP) modulation width of 5 cm, initial nominal range of 16 cm in water (151 MeV), and degraded to 5 cm (78.4 MeV) using 11 cm of solid water plastic placed immediately in front of the mice chambers.

### Blood sample processing procedures

At days 1, 8, 10, 13, 16 and 19 after 2 Gy proton or 2 Gy gamma irradiation, six irradiated mice from each irradiated group and six sham irradiated mice were killed by CO_2_ inhalation, and blood was collected by cardiac puncture and placed into lavender top blood BD microtiner collection tubes containing EDTA (BD, Franklin Lakes, NJ). A total of 90 samples were divided into four aliquots, one aliquot was sent to Antech Diagnostics (Lake success, NY) for complete automated blood count analysis in a Cell-Dyn 3700 multiparameter automated analyzer. The second aliquot was processed immediately after collection, another aliquot was kept at room temperature and processed 24 hours after collection, and the last aliquot was kept at 4°C and then processed 24 hours after collection. For all manual samples, absolute WBC counts and differential counts were determined. Manual absolute WBC counts were determined in a hemacytometer chamber after lysis of red blood cells with 2% acetic acid. For differential counting, blood smears were fixed with 100% methanol for 5 minutes and then stained with Wright-Giemsa stain modified (Sigma Diagnostics, St Louis, MO) according to the manufacturer’s recommendations. Lymphocytes, neutrophils, eosinophils, basophils and monocytes were identified by their staining properties under optical microscopy. A total of 200 cells were counted by an experienced investigator and expressed as the percent of the specific cell type which then was converted to absolute counts utilizing the total WBC counts previously determined. Absolute WBC, lymphocyte and granulocyte (neutrophil + eosinophil + basophil) counts were utilized for comparison with the corresponding sample obtained by automated counts. Since monocytes represent a very small fraction of all mouse blood cells, they were not included in the comparison.

### Statistical analyses

Linear regression and Bland and Altman analysis were utilized to determine correlation and agreement between the two methods. The effects of radiation by manual and automated methods at different times post-irradiation were analyzed by one-way ANOVA followed by the Tukey’s test as well as by a *t*-test. All analyses were done using GraphPad Prism 5 software.

## RESULTS

**Figure 1:**
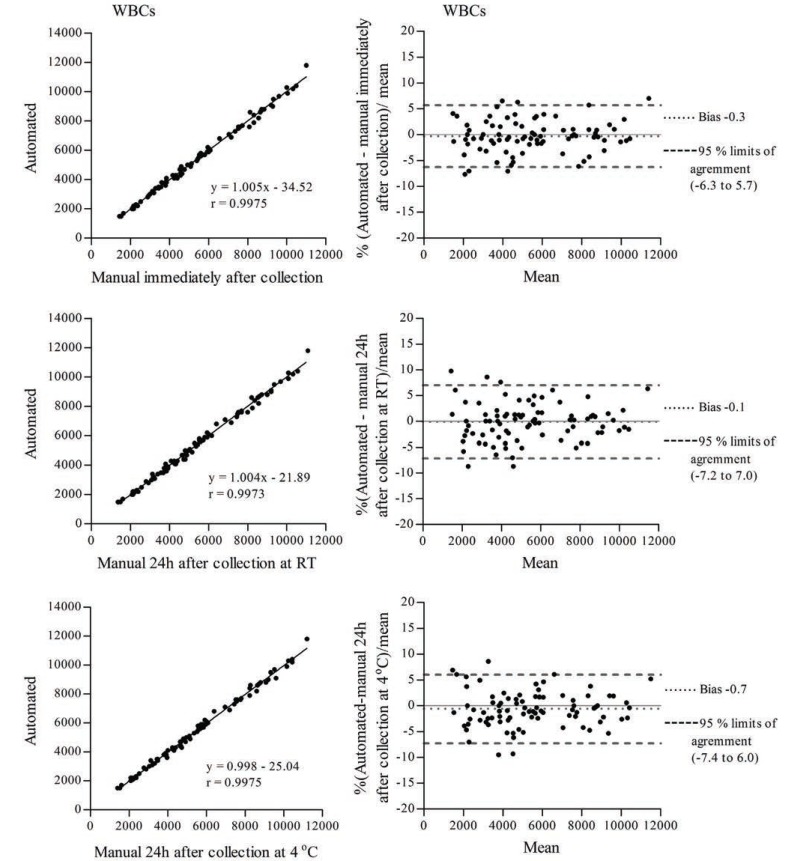
Correlation (left) and Bland and Altman (right) plots of total WBC counts determined between an automated method with samples analyzed up to 24 h after collection and a manual method with samples analyzed immediately after collection (top panel), an automated method with samples analyzed up to 24 h after collection and a manual method with samples analyzed 24 h after collection and stored at RT (middle panel) and an automated method with samples analyzed up to 24 h after collection and a manual method with samples analyzed 24 h after collection and stored at 4°C (bottom panel).

**Figure 2:**
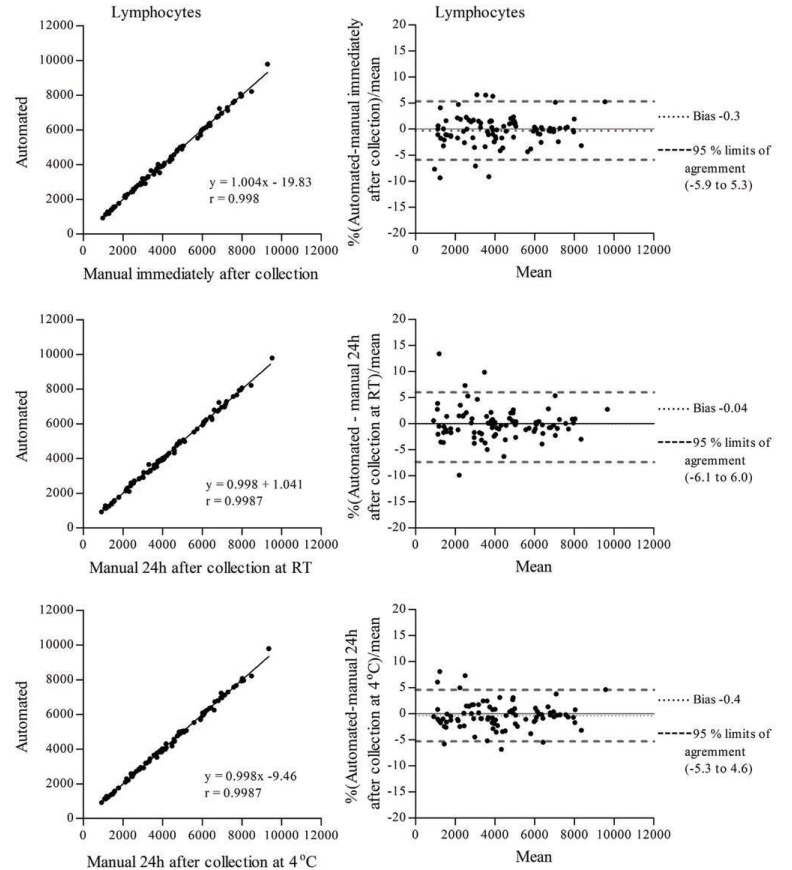
Correlation (left) and Bland and Altman (right) plots of lymphocyte counts determined between an automated method with samples analyzed up to 24 h after collection and a manual method with samples analyzed immediately after collection (top panel), an automated method with samples analyzed up to 24 h after collection and a manual method with samples analyzed 24 h after collection and stored at RT (middle panel) and an automated method with samples analyzed up to 24 h after collection and a manual method with samples analyzed 24 h after collection and stored at 4°C (bottom panel).

**Figure 3:**
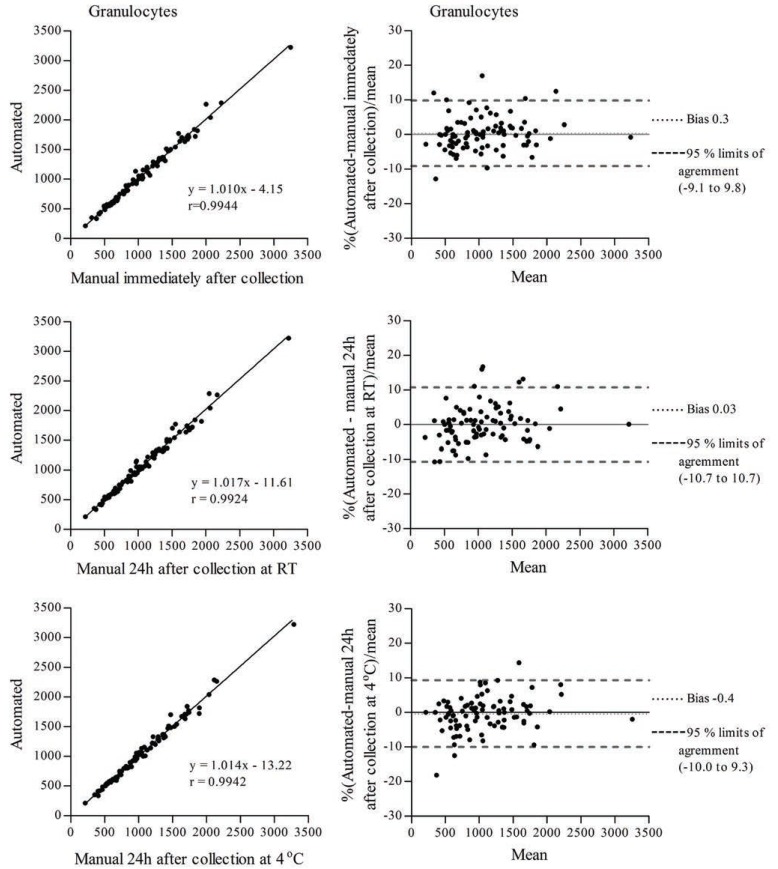
Correlation (left) and Bland and Altman (right) plots of granulocyte counts determined between an automated method with samples analyzed up to 24 h after collection and a manual method with samples analyzed immediately after collection (top panel), an automated method with samples analyzed up to 24 h after collection and a manual method with samples analyzed 24 h after collection and stored at RT (middle panel) and an automated method with samples analyzed up to 24 h after collection and a manual method with samples analyzed 24 h after collection and stored at 4°C (bottom panel).

**Figure 4:**
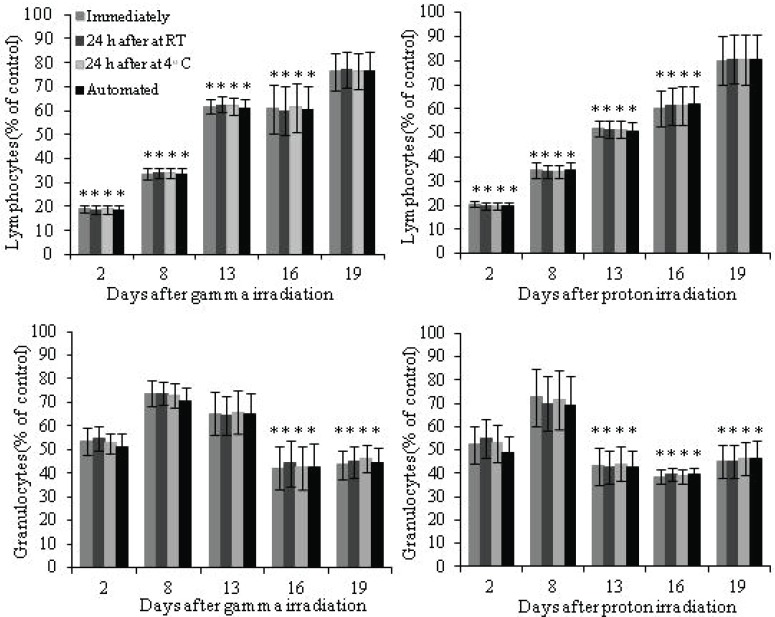
Effect of gamma radiation (left) and proton radiation (right) on lymphocyte counts (top) and granulocyte counts (bottom) determined by a manual method analyzed immediately after collection (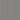), a manual method analyzed 24 hours after collection and stored at RT (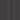) a manual method analyzed 24 hours after collection and stored at 4°C (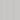) and an automated method analyzed up to 24 h after collection (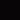). (*p<0.05 by one way ANOVA followed by Tukey’s test of each method compared with its corresponding sham irradiated controls).

To determine the effects of ionizing radiation on blood cells of animal origin, we need to perform blood cell counts in a very large number of samples. Therefore, we decided to utilize an external facility that provides this service. However, we were concerned that our already irradiated blood samples could be affected by the delay in sample processing at the external facility, which may be performed up to 24 h after sample collection. Since it is known that neutrophils (the most abundant granulocyte cells) have a short life span in peripheral blood, 7-10 h in humans (11, 12) and 11.4 h in mice (13) and alterations in WBC counts have been observed in samples stored for prolonged periods of time (10, 14), we decided to conduct a study to directly compare the same irradiated mouse blood samples processed by an external facility with our own manual cell count methods. Additionally, we investigated the effects of the temperature of storage, at room temperature (RT) and at 4°C, although the temperature during sample transportation to the facility of our choice is maintained at 4°C. The comparisons listed in Table 1 were made for total WBCs, lymphocytes and granulocytes. The correlation coefficient (r) for total WBCs, lymphocytes and granulocytes determined by linear regression was r>0.99 for all comparisons (Figures 1, 2 and 3 left panel). Agreement between methods was determined using Bland and Altman analysis. For total WBCs, the 95% limits of agreement for comparison 1 were between -6.3 and 5.7 with a bias of -0.3; for comparison 2, the 95% limits of agreement were between -7.2 and 7.0 with a bias of -0.1 and for comparison 3 the 95% limits of agreement were between -7.4 and 6.0 with a bias of -0.7 (Figure 1 right panel). In the case of lymphocytes, the 95% limits of agreement for comparison 1 were between -5.9 and 5.3 with a bias of -0.3; for comparison 2, the 95% limits of agreement were between -6.1 and 6.0 with a bias of -0.04 and for comparison 3 the 95% limits of agreement were between -5.3 and 4.6 with a bias of -0.4 (Figure 2 right panel). For granulocytes, the 95% limits of agreement for comparison 1 were between -9.1 and 9.8 with a bias of 0.3; for comparison 2, the 95% limits of agreement were between -10.7 and 10.7 with a bias of 0.03 and for comparison 3 the 95% limits of agreement were between -10.0 and 9.3 with a bias of -0.4 (Figure 3 right panel). To determine the effects of ionizing radiation as a function of time on blood cell counts, samples were grouped according to the day they were collected and compared with their corresponding sham irradiated controls; results were expressed as percent of control and are presented in Figure 4 and Table 2. Both 2 Gy proton radiation and 2 Gy gamma radiation caused a statistically significant reduction, of about 80%, in lymphocyte counts two days post-irradiation when compared with corresponding sham irradiated controls, which gradually recovered and by day 19 post-irradiation, the differences between the lymphocyte counts from irradiated animals compared to the corresponding sham irradiated controls were not statistically significant. Granulocyte counts showed a statistically significant decrease (of about 55% of the sham irradiated control values) on days 13, 16 and 19 after proton irradiation and on days 16 and 19 after gamma irradiation. There was no statistically significant difference (*p*>0.05 in all comparisons by *t*-test, Table 2) between the methods (manual and automated) and conditions utilized (analyzed immediately after collection or 24 hours after collection and stored at room temperature or stored at 4°C).

**Table 1:** Comparisons between different conditions of a manual method and an automated method

Comparison	Description

1	Manual counts performed immediately vs. automated counts performed up to 24 hours after sample collection.
2	Manual counts performed 24 hours after collection in samples stored at RT vs. automated counts performed up to 24 hours after sample collection
3	Manual counts performed 24 hours after collection in samples stored at 4°C vs. automated counts performed up to 24 hours after sample collection.

**Table 2:** Statistical analysis of the data presented in Figure 4 as determined by a *t*-test

Days post-irradiation	Comparison	Lymphocytes-*p* value forgamma irradiation	Lymphocytes-*p* value forproton irradiation	Granulocytes-*p* value forgamma irradiation	Granulocytes-*p* value forproton irradiation

2	1	0.9036	0.7843	0.7410	0.7661
	2	0.9682	0.6669	0.9599	0.5986
	3	0.9477	0.8484	0.9747	0.7127
8	1	0.9940	0.6913	0.9961	0.8429
	2	0.8431	0.6640	0.8779	0.9532
	3	0.9384	0.7412	0.9022	0.8891
13	1	0.9337	0.9711	0.8640	0.9517
	2	0.7795	0.9769	0.9019	0.9899
	3	0.9023	0.6455	0.8891	0.877
16	1	0.9940	0.9613	0.8863	0.8375
	2	0.9656	0.9279	0.9577	0.9924
	3	0.9733	0.9665	0.9806	0.8572
19	1	0.9734	0.9170	0.9664	0.9289
	2	0.9619	0.9736	0.9931	0.9049
	3	0.9754	0.8463	0.9826	0.9841

The description of the comparisons is listed in Table 1.

## DISCUSSION

The correlation coefficient obtained for total WBCs, lymphocytes and granulocytes from blood cell samples taken from irradiated mice indicate that there is a strong linear correlation between automated counts and manual counts in all conditions analyzed (immediately after collection or 24 h after collection and stored at RT or 24 h after collection and stored at 4°C). The Bland and Altman analysis shows that the 95% limits of agreement between automated counts and manual counts at all conditions analyzed ranged between -7.4 and 7 for total WBCs; between -6.1 and 6 for lymphocytes and between -10.7 and 10.7 for granulocytes, with almost negligible bias in all cases. Blood cell counts normally vary within a wide range; for instance, in ICR female mice, the reference values for total WBCs have a range of ± 30%, for lymphocytes, the reference values have a range of ± 18% and for granulocytes, the reference values have a range of ± 20%. Therefore, the values obtained from the Bland and Altman analysis are clinically acceptable and demonstrate good agreement between the two methods for all conditions analyzed (immediately after collection or 24 h after collection and stored at RT or 24 h after collection and stored at 4°C), indicating that neither a delay of up to 24 h between sample collection and sample analysis nor storage conditions at room temperature or 4°C had apparent effects on total WBC, lymphocyte or granulocyte counts in blood cell samples taken from irradiated mice. Similar results have been reported in studies utilizing non-irradiated monkey, rabbit, rat, mouse (15) and human blood (16) as well as human synovial fluid (17), in which a delay of 24 to 48 hours showed no adverse effects on WBC counts when EDTA was used as anticoagulant. It has also been reported that EDTA produced the highest yields of neutrophil isolation from human blood when compared with results from studies utilizing heparin or sodium citrate as the anticoagulant (18); therefore, it is likely that in our case, the use of EDTA during collection of blood from the irradiated mice contributed to maintaining the stability of granulocytes as well as of total WBCs and lymphocytes for at least 24 hours after collection. Since a similar pattern was observed in normal human blood samples (16), we expect that the cells in human blood samples taken from patients exposed to radiation would be stable as well. Both 2 Gy proton and 2 Gy gamma radiation caused significant decreases in lymphocyte and granulocyte counts at different times post-irradiation; these changes were determined by both methods to be of comparable magnitudes, with no statistically significant differences between the two methods. To establish a more accurate pattern of the effects of radiation at different days post-irradiation, more studies are required utilizing narrower time points. In summary, our results indicate that radiation-induced effects in blood cells can be accurately determined up to 24 hours after sample collection in samples stored at either RT or 4°C by either manual blood cell counts or by automated blood cell counts. These results suggest that blood cell analyses are not likely to be compromised due to storage time or time spent in travelling to an external facility when the analyses can not be immediately performed on blood cell samples taken from irradiated animals or human patients.
